# Supplementary material for: Morpho-biochemical characterization and molecular marker based genetic diversity of pearl millet (Pennisetum glaucum (L.) R. Br.)
Source: PeerJ. 2023 Jun 5;11:e15403. doi: 10.7717/peerj.15403 (PMC10249620; doi:10.7717/peerj.15403)
Supplement: Table S1 [file peerj-11-15403-s003.docx]

Supp Table 1. List of genotypes and their collection source

| Sr. No. | Name | Source |  | Sr. No. | Name | Source |
| --- | --- | --- | --- | --- | --- | --- |
| 1 | ICMR-06666 | ICRISAT, Hyderabad, India |  | 25 | ICMR-12666 | ICRISAT, Hyderabad, India |
| 2 | ICMR-07222 |  |  | 26 | ICMR-11999 |  |
| 3 | ICMR-08999 |  |  | 27 | ICMR-12777 |  |
| 4 | ICMR-06111 |  |  | 28 | ICMR-09333 |  |
| 5 | ICMR-07888 |  |  | 29 | ICMR-10888 |  |
| 6 | ICMR-08111 |  |  | 30 | ICMR-11777 |  |
| 7 | ICMR-06222 |  |  | 31 | ICMR-12888 |  |
| 8 | ICMR-08666 |  |  | 32 | ICMR-07444 |  |
| 9 | ICMR-06999 |  |  | 33 | ICMR-12999 |  |
| 10 | ICMR-10999 |  |  | 34 | IC-332703 | ICAR-All India Coordinated Millet Improvement Project (AICMIP), Jodhpur, India |
| 11 | ICMR-12333 |  |  | 35 | IC-332715 |  |
| 12 | ICMR-09222 |  |  | 36 | IC-332716 |  |
| 13 | ICMR-10222 |  |  | 37 | IC-332727 |  |
| 14 | ICMR-06555 |  |  | 38 | IC-370523 |  |
| 15 | ICMR-12555 |  |  | 39 | IC-139899 |  |
| 16 | ICMR-07777 |  |  | 40 | IC-139900 |  |
| 17 | ICMR-08222 |  |  | 41 | IC-139903 |  |
| 18 | ICMR-08333 |  |  | 42 | NBPGR-34 |  |
| 19 | ICMR-08444 |  |  | 43 | NBPGR-129 |  |
| 20 | ICMR-09888 |  |  | 44 | GHB-558 |  |
| 21 | ICMR-06888 |  |  | 45 | GHB-905 |  |
| 22 | ICMR-11888 |  |  | 46 | GHB-732 |  |
| 23 | ICMR-07999 |  |  | 47 | Nandi-75 |  |
| 24 | ICMR-12111 |  |  | 48 | 86M-64 |  |
